# High fever following postpartum administration of sublingual misoprostol

**DOI:** 10.1111/j.1471-0528.2010.02564.x

**Published:** 2010-06

**Authors:** J Durocher, J Bynum, W León, G Barrera, B Winikoff

**Affiliations:** a Gynuity Health ProjectsNew York, NY, USA; b Hospital Gineco–Obstétrico Isidro AyoraQuito, Ecuador

**Keywords:** Fever, hyperpyrexia, misoprostol, postpartum haemorrhage (PPH)

## Abstract

**Objective:**

To explore what triggers an elevated body temperature of ≥40.0°C in some women given misoprostol, a prostaglandin E1 analogue, for postpartum haemorrhage (PPH).

**Design:**

*Post hoc* analysis.

**Setting:**

One tertiary-level hospital in Quito, Ecuador.

**Population:**

A cohort of 58 women with a fever of above 40°C following treatment with sublingual misoprostol (800 micrograms) for PPH.

**Methods:**

Side effects were documented for 163 Ecuadorian women given sublingual misoprostol to treat their PPH. Women’s body temperatures were measured, and if they had a fever of ≥40.0°C, measurements were taken hourly until the fever subsided. Temperature trends were analysed, and the possible physiological mechanisms by which postpartum misoprostol produces a high fever were explored.

**Main outcome measures:**

The onset, duration, peak temperatures, and treatments administered for cases with a high fever.

**Results:**

Fifty-eight of 163 women (35.6%) treated with misoprostol experienced a fever of ≥40.0°C. High fevers followed a predictable pattern, often preceded by moderate/severe shivering within 20 minutes of treatment. Body temperatures peaked 1–2 hours post-treatment, and gradually declined over 3 hours. Fevers were transient and did not lead to any hospitalisation. Baseline characteristics were comparable among women who did and did not develop a high fever, except for known previous PPH and time to placental expulsion.

**Conclusions:**

An unexpectedly high rate of elevated body temperature of ≥40.0°C was documented in Ecuador following sublingually administered misoprostol. It is unclear why temperatures ≥40.0°C occurred with a greater frequency in Ecuador than in other study populations using similar treatment regimens for PPH. Pharmacogenetic studies may shed further light on variations in individuals’ responses to misoprostol.

## Introduction

For decades, researchers have explored the most effective, safe, and fast-acting pharmacological agents to manage atonic primary postpartum haemorrhage (PPH), a common cause of excessive bleeding after childbirth. Misoprostol, a prostaglandin E1 derivative, has been investigated as an alternative to conventional parenteral uterotonics for PPH where resources necessary for effective uterotonic (e.g. oxytocin) administration are scarce. Misoprostol is an attractive alternative because of its uterotonic potency, oral administration and stability at ambient temperatures.^1^,^2^ Elevated body temperatures of above 40°C, however, have raised concerns about the safety of this approach.^3^–^5^

The most common side effects associated with the postpartum administration of misoprostol are shivering and pyrexia.^6^ Studies show the rates of shivering and fever to be related, and to be dose- and route-dependent.^5^–^7^ Higher rates of shivering and elevated body temperature are associated with oral and sublingual routes of administration, which achieve a higher and quicker maximum plasma concentration than vaginal or rectal administration.^7^–^9^ One trial comparing 600 micrograms oral versus 600 micrograms rectal misoprostol confirmed that the oral dose resulted in significantly higher rates of shivering (76 versus 54%) and fever (9 versus 1%).^7^ Nevertheless, the reported rates of shivering and fever vary greatly in the literature.^10^ For example, rates of shivering and fever following a prophylactic oral dose of 600 micrograms of misoprostol range from 18 to 71% and from 1 to 38%, respectively.^7^,^11^,^12^ A review of the literature shows that these side effects are not severe and are transient, resolving within 12 hours or less.^1^,^10^,^13^,^14^

In several PPH prevention and treatment studies, misoprostol has been associated with fever of above 40°C (104°F).^12^,^13^,^15^–^19^ One case that called the medical community’s attention to this ‘rare but alarming complication’ involved a reported peak temperature of 41.9°C following 800 micrograms of oral misoprostol given prophylactically.^15^ Other cases of high fever noted in the literature include five of 9198 cases reported from the largest hospital-based clinical trial on the prevention of PPH, in which a prophylactic oral dose of 600 micrograms misoprostol was used.^12^ Four cases of 1026 were reported by Ng and colleagues^13^ after testing a similar regimen. A PPH treatment trial in South Africa reported three women (out of 114) with temperatures of above 40.0°C following 1000 micrograms misoprostol (200 micrograms orally + 400 micrograms sublingually + 400 micrograms rectally).^16^ There have been no other reports of high fever following rectal administration of misoprostol for PPH.^2^,^7^ In Pakistan, a single case of high fever (out of 29) following adjunct treatment with a sublingual dose of 600 micrograms was reported.^17^ More recently, two multicentre studies testing an 800 micrograms regimen of sublingual misoprostol as first-line treatment for PPH reported a higher-than-expected rate of fever above 40°C in one of nine sites (36%), whereas much lower rates were recorded in the other eight sites, ranging from 0 to 9%.^18^,^19^ In all of these hospital-based reports, the elevated temperatures did not result in further health complications.

Reports of fever of ≥40.0°C following misoprostol for PPH have been described on separate occasions as cases of hyperthermia^15^ and severe pyrexia or hyperpyrexia.^5^,^6^,^16^ These two terms used to describe high fevers actually imply very different biologic mechanisms. Hyperpyrexia results from a regulated upward shift in the hypothalamic set point, which triggers the body to conserve and produce heat to attain the new set point.^20^ In contrast, hyperthermia occurs when temperature increases in the absence of a shift in hypothalamic set point^21^,^22^: heat conservation measures (e.g. shivering and seeking warm places) are not induced, and temperature elevation occurs in an unregulated manner, making it particularly dangerous. Hyperthermia is relatively rare compared with hyperpyrexia; nonetheless, what triggers elevated temperature in some women following misoprostol administration remains unconfirmed.

This manuscript presents a review of high fevers (of ≥40.0°C) occurring at one hospital in Quito, Ecuador, following the administration of 800 micrograms sublingual misoprostol for PPH treatment. A detailed analysis of the temperature trends of high fevers that occurred at this high altitude (2,800 m) site is followed by a discussion of the possible physiological mechanisms by which postpartum misoprostol administration produces fever.

## Methods

Two large clinical trials were conducted to evaluate the efficacy, safety, and acceptability of sublingual misoprostol (800 micrograms) as a first-line treatment of PPH among women undergoing vaginal delivery with suspsected uterine atony.^18^,^19^ The sublingual route was identified as having the greatest potential for the treatment of PPH because of its rapid uptake, long-lasting duration of effect, and greatest bioavailability, compared with other routes of misoprostol administration.^9^ The studies compared misoprostol with oxytocin using a randomised, double-blind placebo-controlled non-inferiority study design. In total, 1787 women were treated with one of two regimens: 800 micrograms of sublingual misoprostol (*n* = 895) plus one ampoule of saline solution or 40 iu of intravenous (IV) oxytocin (*n* = 892) plus placebo tablets resembling misoprostol. Providers and women were masked to treatment assignment. Measured postpartum blood loss, change in pre- to post-delivery haemoglobin levels, and recourse to additional interventions beyond the initial study treatment were documented to assess the efficacy of each uterotonic therapy. The median blood loss at the time of PPH treatment was 700 ml, and active bleeding was controlled within 20 minutes with initial study treatment alone for nine out of ten women treated with misoprostol. Hospitals from Burkina Faso, Ecuador, Egypt, Turkey, and Vietnam participated in the clinical research from August 2005 until January 2008. The study findings and trial design have been reported separately.^18^,^19^

The present study is a *post hoc* analysis of the side effect profiles and the acceptability of secondary effects associated with misoprostol treatment. Data were collected on the maternal side effects detected by providers or reported by women within the first 20 minutes of treatment administration. Delivery attendants rated the severity (mild, moderate or severe) of any side effect noted, and recorded any treatment given to manage it. Side effects necessitating treatment were managed according to each hospital’s clinical protocol. If fever was perceived by women or delivery attendants, body temperature was then measured with the standard thermometers routinely used in each centre. At 3 hours postpartum, delivery attendants reassessed the health status of the women and recorded any side effects that were experienced since the last observation. Prior to discharge from the hospital, delivery attendants interviewed women about the acceptability of side effects following treatment.

Investigators at all sites were informed of any unexpected or excessively frequent side effects, and any serious adverse events occurring at other sites. An independent Data Safety and Monitoring Board was established to review reports of adverse events, provide advice on risk management, and review interim analyses to ensure the continued scientific validity and merit of the study. Regular monitoring and retraining of the delivery ward staff continued throughout the duration of the trial. The study protocol was approved by the Western Institutional Review Board (WIRB) as well as all relevant local ethics committees.

Following the first report of a body temperature of ≥40.0°C, delivery attendants were retrained on how to recognise, measure, and manage fever. Management practices for reducing fever included removing blankets from the patient, applying cool compresses, administering oral acetaminophen, and ensuring adequate hydration by mouth or IV. To capture the details associated with this side effect in Ecuador, where reports of high fever were most frequent, delivery attendants were asked to complete an additional study form to document the onset, duration, peak temperatures, and treatment of cases with high fever. When fever was observed, delivery attendants measured the woman’s body temperature, and continued to measure her temperature at a maximum of hourly intervals using an oral mercury thermometer, until the fever subsided (measuring below 38.0°C). Tympanic and digital oral thermometers were also used to compare results with the oral mercury thermometer.

These additional study forms were entered into a separate database, merged with Ecuadorian data from the larger trial, and analysed using the Statistical Package for the Social Sciences v13.0 (SPSS, Chicago, IL, USA). Descriptive statistics were calculated for maternal side effects and their severity. An elevated body temperature measuring ≥40.0°C or 38–39.9°C were classified as high or mild/moderate fever, respectively. Comparisons between Ecuadorian women with and without high fever were performed using chi-square and Fisher’s exact tests. Subgroup analyses were conducted to establish consistency of efficacy and safety for various subgroups or risk groups. Rates of high fever in Ecuador were compared with reported rates from other sites. Relative risk (RR) and 95% confidence intervals were calculated as appropriate.

## Results

A total of 895 women received 800-micrograms of sublingual misoprostol for the treatment of primary PPH. The most prevalent side effects following misoprostol treatment were shivering (42.6%; 381/895) and fever (34.1%; 305/895). Reports of shivering, fever, and temperature of ≥40.0°C among women receiving misoprostol varied across sites (Table 1). At the hospital in Ecuador, 35.6% (58/163) of women receiving misoprostol had a fever of ≥40.0°C, compared with reported rates that ranged from 0 to 9.5% in the other eight hospitals. There were no reports of side effects resulting in any prolonged hospital stay, and all women with high fever made a full recovery.

**Table 1:** Rates of shivering, fever, and temperature ≥40.0°C by site following sublingual misoprostol for PPH treatment

	Any shivering		Any fever		Fever ≥ 40.0°C	
	*n*/*N*	%	*n*/*N*	%	*n*/*N*	%
**Ecuador**						
Quito*	146/163	89.6	151/163	92.6	58/163	35.6
**Burkina Faso**						
Bobo Dioulasso**	22/34	64.7	10/34	29.4	0/34	0.0
**Egypt**						
Alexandria*	14/198	7.1	9/198	4.5	0/198	0.0
Cairo**	58/236	24.6	48/236	20.3	3/236	1.3
**Turkey**						
Ankara**	25/33	75.8	14/33	42.4	1/33	3.0
**Vietnam**						
Binh Duong*	31/53	58.5	15/53	28.3	1/53	1.9
Tu Du*	38/74	51.4	42/74	56.8	7/74	9.5
Cu Chi**	28/52	53.8	13/52	25.0	0/52	0.0
Hoc Mon**	19/52	36.5	3/52	5.8	1/52	1.9

* These sites participated in the PPH treatment trial conducted among women not exposed to oxytocin during labour.^19^

** These sites participated in the PPH treatment trial conducted among women who received oxytocin prophylactically.^18^

**Figure 1:**
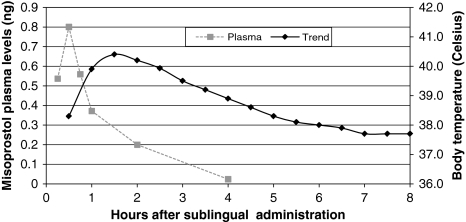
Mean misoprostol plasma concentrations after sublingual administration of misoprostol (800 micrograms),^23^ and mean temperatures of 58 cases of high fever over time.

Temperature trends were documented for the 58 cases of high fever (≥40.0°C) in Quito, Ecuador. High fever was typically characterised by a sharp increase in temperature within 1 hour of treatment, a peak in temperature 1–2 hours post-treatment, and a gradual decline in temperature over a period of 3 hours. Average temperatures remained above 40.0°C for less than 2 hours, and measured below 38.0°C approximately 6 hours after receiving misoprostol (Figure 1). Temperature trends for mild/moderate fevers followed a similar pattern, but with lower peak temperatures (data not shown). Women with high fever were treated with oral acetaminophen, cool compresses, and aspirin delivered intravenously. Treatment practices were similar for women with mild to moderate fevers, although a subset of women with mild/moderate fevers did not receive treatment with IV aspirin. An analysis of this subset showed that cases of mild to moderate fevers treated with IV aspirin resolved similarly to those that were treated with only oral acetaminophen and/or cold compresses (data not shown).

In Ecuador, almost all participants (92.6%; 151/163) receiving 800 micrograms of misoprostol sublingually experienced an elevated body temperature (≥38.0°C). Shivering usually accompanied fever, irrespective of peak temperature (89.6%; 146/163). Severe shivering (defined as uncontrollable shaking that made it difficult to articulate or control physical movement) was more frequently reported among women with temperatures of ≥40.0°C (27.6%; 16/58) compared with those without (2.9%; 3/105; RR 9.66; 95% CI 2.94–31.8). Moderate shivering was described as producing strong trembling that did not affect speech or mobility. High fevers were often preceded by moderate or severe shivering within the first 20 minutes of receiving misoprostol [41.4% (24/58) versus 13.3% of women who did not develop high fever (14/105); RR 3.10; 95% CI 1.74–5.52]. Other known side effects of misoprostol, such as nausea, vomiting, and diarrhoea, were infrequent, and rates did not vary by degree of fever. Transient delirium or altered sensorium (such as disorientation, confusion, decreased bilateral/blurry vision, speech impairment, muscular stiffness, neuromotor agitation, or hallucinations) was reported in eight women (8/58) with high fever versus three women (3/93) who had mild/moderate fever (RR 4.28; 95% CI 1.18–15.5).

Baseline characteristics were comparable among Ecuadorian women who did and did not develop a high fever, except for previous PPH and rapid placental expulsion (Table 2). Outcomes associated with postpartum blood loss in Ecuador (i.e. efficacy of initial uterotonic therapy, time to control active bleeding, and total blood loss) did not vary between women with high fever and those without, demonstrating consistency in treatment outcomes among subgroups. Recourse to additional interventions (including blood transfusion, exploration under anaesthesia, and the administration of additional uterotonics) was similar among women with high fever and those without. Among the women who were given additional uterotonics (*n* = 12), rectal misoprostol (200 micrograms) was given to one woman in the high fever group (1/4), and to one woman in the no high fever group, who received 800 micrograms (1/8). 48.3% of women (28/58) who developed high fever were administered IV fluids/electrolytes following temperature elevation, compared with 35.2% (37/105) of women without high fever (*p* = 0.072). Women who developed high fever were as likely to be reported in ‘good’ condition at discharge as were those who did not experience high fever.

**Table 2:** Characteristics of Ecuadorian population by incidence of high fever following misoprostol treatment

	Developed high fever *n* = 58	No high fever *n* = 105	*P* value
**Age (years)**			
Younger than 20	17 (29.3)	32 (30.5)	0.076
20–34	40 (69.0)	61 (58.1)	
35 or older	1 (1.7)	12 (11.4)	
**No. of previous live births**			
0	24 (41.4)	41 (39.0)	0.829
1–3	29 (50.0)	57 (54.3)	
4+	5 (8.6)	7 (6.7)	
**Pre-delivery haemoglobin less than 11.5 g/dL**	6 (10.3)	3 (2.9)	0.053
**Gestational age (weeks)**			
Pre-term (less than 37)	1 (1.7)	5 (4.8)	0.241
Term (37.0–40.9)	47 (81.0)	90 (85.7)	
Post-term (41 or more)	10 (17.2)	10 (9.5)	
**Known previous PPH**	6 (10.3)	2 (1.9)	0.024
**Multiple pregnancy**	0 (0.0)	0 (0.0)	–
**Oxytocin given in third stage of labour or earlier**	0 (0.0)	0 (0.0)	–
**Epidural given**	2 (3.4)	3 (2.9)	0.585
**Suturing after delivery**	39 (67.2)	79 (75.2)	0.274
**Placental delivery within 15 minutes**	48 (82.8)	69 (65.7)	0.021
**Blood loss (ml) at time of treatment (median IQR)**	850 (750–950)	850 (750–1000)	0.322

Numbers are *n* (%) unless otherwise specified.

On average, women were discharged from the hospital in Ecuador approximately 27.4 (±9.9) hours following delivery, independent of the incidence of fever (*P*= 0.749). Exit interviews were conducted immediately prior to discharge for all women. Among those women who had fever of ≥40.0°C, one-third (18/58) did not report having experienced this side effect; likewise, no provider reports of delirium/altered sensorium or fainting were confirmed by women during the exit interviews. Nevertheless, 50.9% (55/108) and 44.0% (44/100) of women who confirmed provider reports of having experienced shivering and fever during their exit interviews characterised them as being ‘intolerable’. Women who experienced high fevers were more likely to report shivering as ‘intolerable’, compared with women who did not develop high fever (RR 1.51; 95% CI 1.04–2.18). Interestingly, women who developed high fever were no more likely than those who experienced mild/moderate fevers to report the fever itself as ‘intolerable’ (RR 1.50; 95% CI 0.97–2.32).

## Discussion

These results confirm that women who receive misoprostol postpartum are at risk for shivering and fever. As in previous studies, such effects were related, transient, self-limiting, and did not result in additional health complications.^1^,^10^,^13^–^15^ Nevertheless, a striking finding was the unexpected rate of high fever in Ecuador as compared with other study sites, with over one-third of women treated with misoprostol developing a temperature of ≥40.0°C. A comparison of the overall rates of shivering and fever between sites shows that the thermoregulatory response to misoprostol among Ecuadorian women is notably different from women treated at the other sites (Table 1).

Prior to the present study, the detailed documentation of fever characteristics, particularly fevers measuring ≥40.0°C, has been scant.^12^,^15^–^17^ The temperature trends recorded in our study show misoprostol-induced fevers followed a predictable pattern, and high fevers were often preceded by moderate or severe shivering within the first 20 minutes of misoprostol administration. In contrast to the rapidly fatal, irregular, uncontrolled spikes in temperature associated with hyperthermia, high fever in Ecuador exhibited a distinctive, consistent pattern: temperatures peaked approximately 1.5 hours post-sublingual misoprostol administration, and decreased thereafter. As shown in Figure 1, the pattern of temperature elevation mimics misoprostol blood plasma concentration following sublingual administration.^23^ The 30–60-minute lag between the peaks in plasma concentration and temperature may be attributable to the time it takes for the febrile signal to be received and processed in the hypothalamus, as well as for the physiological processes associated with fever to elevate the body temperature.^24^ These data suggest that the temperature elevation associated with misoprostol use is dependent on plasma concentrations, and explains why fever is dose- and route-dependent.^5^–^7^

The temperature elevations associated with misoprostol are compatible with a shift in the hypothalamic set point, and do not appear to be cases of hyperthermia, but rather of pyrexia. Indeed, E-series prostaglandins (PGEs) are involved in the endogenous fever mechanism, and prostaglandin E2 (PGE_2_) in particular is acknowledged as the primary mediator of fever induction^20^ through an interaction with the EP3 receptor.^25^,^26^ However, there is no evidence that prostaglandin E1 (PGE_1_), of which misoprostol is an analogue, acts differently from PGE_2_:^20^,^27^ in fact, the biologically active form of misoprostol, misoprostol acid, has been shown to bind to the EP3 receptor.^28^ Considering this evidence, we theorise that in the fever cases presented, misoprostol may be mimicking endogenous PGEs in the thermoregulatory pathway by shifting the hypothalamic set point upwards and stimulating temperature elevation. Further pharmacologic studies are needed to validate this hypothesis. Importantly, these fevers were well managed by nurses with local treatment practices within the clinical competencies of delivery attendants. Fevers followed a predictable course (Figure 1), and it is not clear if treatment practices had any effect on fever resolution. Because antipyretics work by inhibiting endogenous prostaglandin production,^24^ the lack of treatment effect appears consistent with fever resulting from exposure to exogenous prostaglandins.

Some researchers have suggested that the increased rate of fever following postpartum misoprostol administration may result from a lowered threshold for prostaglandin-induced temperature elevation in term pregnant women.^29^ However pre-clinical work suggests that, conversely, term pregnancy naturally suppresses fever because of an increase in the endogenous production of antipyretics and a decrease in endogenous pyrogen formation.^30^ Rates of fever following misoprostol treatment in postpartum women at term however, do seem to exceed rates in women given similar doses earlier in gestation.^31^

Despite the uncertain relationship between prostaglandins, gestation, and fever, it is well known that endogenous prostaglandins play a role in the physiological processes involved in labour and delivery. Prostaglandins are produced by the intrauterine tissues and are involved in the rupture of the membranes, cervical ripening, myometrial contractility, placental separation, and uterine involution.^28^,^32^ In fact, postpartum shivering is not uncommon,^33^ and may be related to the release of prostaglandins at parturition. In the present study, high fever was more common among Ecuadorian women who experienced a rapid expulsion of the placenta (Table 2). Because endogenous prostaglandins are involved in placental separation,^32^ the concurrent flood of both endogenous and exogenous prostaglandins may have increased the risk of shivering and fever in Ecuador. Interestingly, placental size is typically larger in high-altitude populations (a developmental response to the hypoxic environment), and should be considered further by researchers studying these physiological processes.^34^

Few PPH studies testing oral or sublingual misoprostol regimens have systematically measured body temperature at predetermined time intervals following postpartum use.^7^,^10^,^13^,^14^,^17^,^35^ Some studies have documented the occurrence of fever based on routine temperature measurement at 1-hour postpartum; others have measured temperature only after fever was reported by women or detected by attendants. These methods may have resulted in the under-reporting of fever in previously published studies, as well as in the study sites discussed in this manuscript. The level of postpartum monitoring may also lead to varying rates and/or under-reporting of fever across hospitals participating in the same protocol. Because the onset of moderate/severe shivering and high fever following misoprostol administration for PPH is visibly detectable by the delivery attendant within the first hour post-treatment, we do not believe that the differences in the reports of high fevers among hospitals are attributable to variation in duration of hospital stay. Furthermore, following the first reports of elevated body temperature in Quito, study teams at the other sites were alerted to the possibility of such effects. Regular monitoring visits to these sites confirmed that the occurrence of shivering, fever, and temperatures of ≥40.0°C remained consistent at all sites for the duration of the study. Efforts to call the study team’s attention to the possibility of these effects did not result in increasing reports of high fevers.

It is unclear why women in Quito demonstrated uncharacteristically high rates of fever following postpartum administration of misoprostol. Following the first reports of high fever, the study team in Ecuador reviewed both their clinical practices and the patient characteristics that could possibly contribute to the increased rate of high fevers. Patterns or associations with other medications taken and/or other health conditions were explored, and none were identified. At study completion, the treatment arms were unmasked, revealing that high fever only occurred among women treated with misoprostol; no cases with high fever were documented in the oxytocin group in any study site. The occurrence of high fevers was also found to be evenly distributed over the course of the study, and not clustered around a specific time frame, which might have indicated a problem with infection in the labour or delivery ward, or with the study supplies. Given that high fever only occurred among women treated with misoprostol, was short-lived, and was not treated with antibiotics, infection is not suspected to be the cause.

Environmental factors such as Quito’s high elevation as well as genetic factors were also explored. It may be that a genetic variation permits misoprostol, a PGE_1_ analogue, to activate the endogenous fever mechanism that is typically triggered by PGE_2_. If this is the case, the high rate of fever among Ecuadorian women may represent a high frequency of a variant allele in this homogenous population. The potential role of altitude on fever incidence also remains speculative. In fact, there have been no reports to date of high fevers occurring after misoprostol administration in the puerperium in other high-elevation settings.^36^,^37^ Other environmental factors such as the ambient temperature in Quito are not suspected to have contributed to the rate of high fevers. Because of its elevation and its proximity to the equator, Quito has a fairly constant cool climate, with an average year-round temperature of 19°C (66°F).

Importantly, the participant population in Quito was highly homogenous; therefore, the incidence, treatment, and cause of the high fevers presented in this paper may have limited generalisability to other populations. Apart from the study sites discussed in this paper, it is not known whether other populations will also experience similar rates of high fever.

Although there are many questions that remain about the incidence of high fever in Ecuador, our findings concur with previous experience and research that have shown the side effects following misoprostol administration not to be life-threatening.^1^,^10^,^38^ Fever is commonly observed when misoprostol is given for a range of health indications. The temperature trends documented in this study provide reassurance to clinicians that misoprostol-induced fevers (regardless of how high the peak temperature) are transitory. Lower dosages or different routes of administration may minimise the occurrence of such events.^3^,^6^ Currently, however, no data support other routes of administration or lower doses of misoprostol as a first-line treatment for PPH.^2^,^18^,^19^ Furthermore, we do not know if treatment affects the course of misoprostol-induced fevers: the cases of fever presented in this paper followed a predictable pattern seemingly independent of the type of management. Nevertheless, providers should be informed of what to expect regarding body temperature elevation, shivering and other side effects following postpartum misoprostol administration, and should be advised of acceptable treatment and palliative measures.

Until definitive relationships between genetic or environmental variation and drug response can be established, the questions of why some women develop high body temperature, and why so many high fevers occurred in Ecuador, remain. The recent burgeoning of pharmacogenetic studies may shed light on these hypotheses.
